# From more testing to smart testing: data-guided SARS-CoV-2 testing choices, the Netherlands, May to September 2020 

**DOI:** 10.2807/1560-7917.ES.2022.27.8.2100702

**Published:** 2022-02-24

**Authors:** Janko van Beek, Zsofia Igloi, Timo Boelsums, Ewout Fanoy, Hannelore Gotz, Richard Molenkamp, Jeroen van Kampen, Corine GeurtsvanKessel, Annemiek A van der Eijk, David van de Vijver, Marion Koopmans

**Affiliations:** 1 Department of Viroscience, ErasmusMC, Rotterdam, the Netherlands; 2 Department Infectious Disease Control, Public Health Service Rotterdam-Rijnmond, Rotterdam, The Netherlands

**Keywords:** SARS-CoV-2, rapid detection test, infectiousness, public health policy

## Abstract

**Background:**

SARS-CoV-2 RT-PCR assays are more sensitive than rapid antigen detection assays (RDT) and can detect viral RNA even after an individual is no longer infectious. RDT can reduce the time to test and the results might better correlate with infectiousness.

**Aim:**

We assessed the ability of five RDT to identify infectious COVID-19 cases and systematically recorded the turnaround time of RT-PCR testing.

**Methods:**

Sensitivity of RDT was determined using a serially diluted SARS-CoV-2 stock with known viral RNA concentration. The probability of detecting infectious virus at a given viral load was calculated using logistic regression of viral RNA concentration and matched culture results of 78 specimens from randomly selected non-hospitalised cases. The probability of each RDT to detect infectious cases was calculated as the sum of the projected probabilities for viral isolation success for every viral RNA load found at the time of diagnosis in 1,739 confirmed non-hospitalised COVID-19 cases.

**Results:**

The distribution of quantification cycle values and estimated RNA loads for patients reporting to drive-through testing was skewed to high RNA loads. With the most sensitive RDT (Abbott and SD Biosensor), 97.30% (range: 88.65–99.77) of infectious individuals would be detected. This decreased to 92.73% (range: 60.30–99.77) for Coris BioConcept and GenBody, and 75.53% (range: 17.55–99.77) for RapiGEN. Only 32.9% of RT-PCR results were available on the same day as specimen collection.

**Conclusion:**

The most sensitive RDT detected infectious COVID-19 cases with high sensitivity and may considerably improve containment through more rapid isolation and contact tracing.

## Background

By 3 February 2022, more than 90 million coronavirus disease (COVID-19) cases and 960,000 related deaths had been reported in the European Union and European Economic Area (EU/EEA) and it has been challenging for public health authorities to keep up the test, trace and isolate (TTI) strategy [1]. The purpose of this TTI strategy is to stop transmission chains and reduce the impact of COVID-19. Epidemiological modelling suggests that aggressive TTI combined with physical distancing, and the use of personal protective equipment when physical distancing is not achievable, could suppress virus transmission below a level that exceeds hospital capacity without need for a lockdown [2]. Case diagnosis based on RT-PCR testing has limitations in terms of time to result, and scaling up of test capacity has been hampered in the past by scarcity of critical reagents during the early phase of the pandemic. In addition, while highly specific, the sensitivity of RT-PCR combined with prolonged shedding of small amounts of viral RNA for weeks may lead to positive test results following clinical recovery long after a person is infectious [3]. Furthermore, screening of persons without symptoms may yield weak positive test results, raising questions about how to handle such cases. Ideally, screening of cases would be based on testing for infectivity, but cell culture-based assays, used as a proxy for infectivity, have long turnaround times and are therefore not suitable for rapid screening. Rapid antigen detection tests (RDT) have entered the diagnostic market. Compared with RT-PCR, they are relatively easy to produce, easy to use with faster turnaround times, and, depending on the assay, without the need for dedicated equipment or high-level laboratory capacity. The widespread and frequent use of such tests has been proposed as a solution to the safe reopening and return to the pre-pandemic social interactions, but only limited information is available on the ability of RDT to detect infectious cases [4]. Here, we assess the potential impact of introduction of RDT in the current test strategy of the Netherlands where the majority of testing is done in drive-through test stations.

## Methods

### Patients and metadata

A database of the public health service Rotterdam-Rijnmond with RT-PCR-confirmed COVID-19 cases detected between 26 May and 5 September 2020 was linked with the laboratory database with viral load data of the Erasmus MC to assess the relationship between viral load and days after disease onset.

### Sample collection and national SARS-CoV-2 testing guidelines

Nasopharyngeal/throat specimens were obtained by trained personnel at a COVID-19 test facility and specimens were sent to the Erasmus MC on the same day. Testing for severe acute respiratory syndrome coronavirus 2 (SARS-CoV-2) was provided free of charge to Dutch residents with COVID-19-like symptoms or with recent contact with a confirmed case. At the time of study, children younger than 7 years were not advised to test, and children between 7 and 11 years-old were only tested if they had fever, chest tightness, or recent contact with a confirmed case.

### Diagnostic testing and calculation of genomic copy number 

Routine RT-PCR testing was performed on combined nasopharyngeal and throat swabs in virus transport medium using the Cobas SARS-CoV-2 test on the COBAS6800 (Roche diagnostics, Basel, Switzerland). We translated quantification cycle (Cq) values to log10 RNA copies/mL using calibration curves based on quantified in vitro RNA transcripts of the E gene [5]. Turnaround time of the RT-PCR testing was logged systematically at the Erasmus MC to assess a potential impact of the implementation of antigen testing on the time to result.

### Virus isolation

The SARS-CoV-2/NL/2020 isolate (https://www.european-virus-archive.com/virus/sars-cov-2-strain-nl2020) was propagated by inoculation on Vero E6 cells and harvested after 48 h. The preparation was not further purified in order to mimic clinical samples, which contain a combination of released virus particles and cell lysate. The virus stock was titrated in a 10-fold serial dilution and inoculated on Vero E6 cells with visual inspection for cytopathic effect (CPE) after 48–72 h (expressed as median tissue culture infectious dose (TCID50)). Sequencing of the virus stock was performed to exclude cell line adaptations. Seventy-eight freshly obtained specimens (no freeze/thaw steps) were tested by RT-PCR and culture to determine RNA loads in relation to TCID50 titres of infectious virus. Vero cell clone 118 was used for isolation of infectious SARS-CoV-2 from respiratory tract samples to allow more rapid growth compared with the Vero E6 cell line. Samples were cultured for 14 days and the presence of SARS-CoV-2 was confirmed with immunofluorescent detection of nucleocapsid proteins.

### Antigen rapid tests

We chose five RDT based on availability and performance as claimed by the manufacturer: Panbio COVID-19 Ag rapid test (Abbott, Chicago, United States, Lot number: 41ADF011A), Standard Q COVID-19 Ag (SD Biosensor, Inc, distributed by Roche, Lot number: QCO3020079/Sub A-2), COVID-19 Ag Respi-Strip (Coris BioConcept, Gembloux, Belgium, Lot number: 42969D2016), GenBody COVID-19 Ag (GenBody Inc, Cheonan, South Korea, Lot number: FMFY03201) and Biocredit COVID-19 Ag (RapiGEN Inc, Gunpo, South Korea, Lot number: H073011SD). All RDT were colloidal gold lateral flow strips using the nucleocapsid protein of SARS-CoV-2 as antigen target. None of the RDT requires dedicated laboratory equipment and all RDT can be performed in the field. We assessed the analytical limit of detection (LoD) of the RDT by testing a 10-fold serial dilution of the cultivated SARS-CoV-2 from frozen stock with known viral RNA concentration in triplicates. The recommended amount (100 or 130 μL) of samples were diluted 1:1 in the relevant reaction buffer and added to the tests according to the manufacturer’s instructions. Results were read out visually after the manufacturer’s recommended incubation time of 15 to 30 min.

### Data analyses

Logistic regression analysis using R version 4.0.0 (R Foundation, Vienna, Austria) was used to determine the association between virus-positive culture success and 10log-transformed viral RNA load. The projected probability for viral infectivity given the viral load was calculated by converting the log odds as determined in the logistic regression analysis to a probability for viral culture success. We calculated the projected viral isolation success for every viral RNA load found at the time of diagnosis in 1,754 specimens of confirmed cases diagnosed with SARS-CoV-2 infection presenting at a drive-through test station. The number of individuals with a projected viral isolation success was calculated as the sum of the probabilities of viral isolation success times the number of people presenting within a particular viral load range above the LoD of the various RDT.

## Results

**Figure 1:**
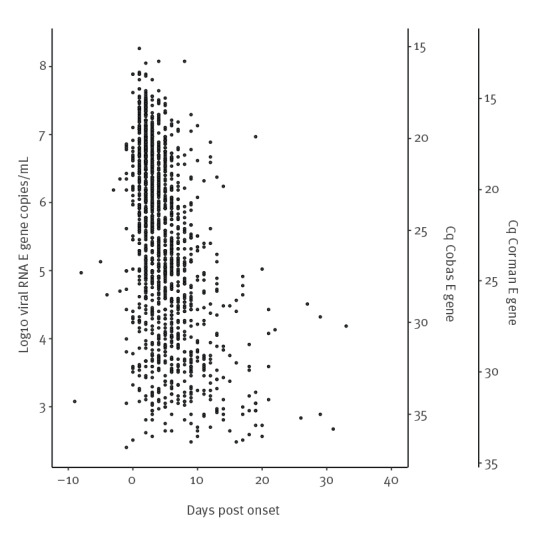
SARS-CoV-2 RNA load by time since onset for RT-PCR-positive samples at a drive-through test station, Rotterdam, the Netherlands, 26 May–5 September 2020 (n = 1,450^a^)

A total of 1,739 confirmed COVID-19 cases were found with 1,754 RT-PCR-positive specimens between 26 May and 5 September 2020. The median age of cases was 35 years (range: < 1 to 93 years) and 913 of 1,733 (52.7%) cases were female (gender of six cases not registered). Of 1,450 cases with known onset date, 1,096 (75.6%) persons had reported to the testing station within the first week of symptom onset, in line with national recommendations for testing (Figure 1).

Cq: quantification cycle.

^a^ RNA load of 304 specimens with unknown symptom onset date are not shown in this figure.

Right y-axis shows Cq values for the E gene target of two commonly used RT-PCR platforms: (Cobas SARS-CoV-2 test, Roche Diagnostics and Corman et al. PCR [14]). Viral loads of nine samples were outside plot boundaries and not plotted in this figure.

**Figure 2:**
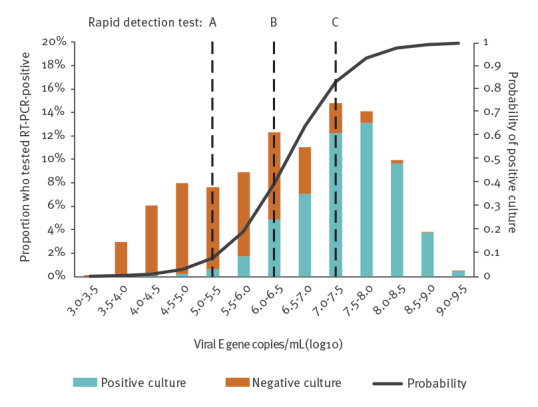
Viral RNA loads at time of diagnosis of community cases with RT-PCR-confirmed SARS-CoV-2 infection presenting to a drive through test station, Rotterdam, the Netherlands, 26 May–5 September 2020 (n = 1,754^a^)

^a^ 1,754 specimens of 1,739 cases.

The distribution of Cq values and estimated viral RNA loads for patients reporting to drive-through testing was skewed to high viral RNA loads (Figure 2). The relationship between viral load and infectiousness of patients with mild disease symptoms was assessed by culturing fresh specimens obtained from 78 randomly selected individuals (median age: 38.5 years, range: 13–82 years, 43/78 (55%) female) who were diagnosed with SARS-CoV-2 infection in the drive-through testing station. We determined the probability of being infectious based on the viral RNA load for all patients tested in the drive-through station using logistic regression analysis. We used this analysis to calculate the density distribution of infectious individuals (Figure 2). In addition, we calculated infectiousness based on two published studies that tested cell culture in parallel with RT-PCR in hospitalised patients with severe and mild illness (Table). In addition, we provide the density distribution of mild and severe hospitalised infectious individuals in Supplementary Figure S1) [3,6].

Black line: probability of having infectious virus in the nose/throat swab based on logistic regression analysis of culture test results of 78 freshly obtained specimens with known viral load of community cases. Blue bars: predicted number of cases with shedding of infectious virus at time of diagnosis (positive culture); Brown bars: number of predicted cases without shedding of infectious virus at time of diagnosis (negative culture). Dashed vertical lines: limit of detection of the different rapid detection tests: A: PanbioTM COVID-19 Ag rapid test (Abbott), and Standard Q COVID-19 Ag (SD Biosensor); B: COVID-19 Ag Respi-Strip (Coris BioConcept), and GenBody COVID-19 Ag (GenBody Inc); C: Biocredit COVID-19 Ag (RapiGEN).

**Table:** Estimated proportion of detected culture-positive samples of RT-PCR SARS-CoV-2-confirmed samples by rapid antigen tests with different detection limits, Rotterdam, the Netherlands, 26 May–5 September 2020 (n = 1,754 specimens)

Rapid antigen assay group	Mild, outpatient median (min–max)	Hospitalised, mild median (min–max)	Hospitalised, severe median (min–max)
A	97.30% (88.65–99.77)	98.68% (95.79–99.81)	99.80% (99.32–99.97)
B	92.73% (60.30–99.77)	97.43% (86.40–99.81)	99.54% (97.45–99.97)
C	75.53% (17.55–99.77)	91.70% (57.90–99.81)	98.55% (88.53–99.97)

Group A: Panbio COVID-19 Ag rapid test (Abbott) and Standard Q COVID-19 Ag (SD Biosensor); Group B: COVID-19 Ag Respi-Strip (Coris BioConcept) and GenBody COVID-19 Ag (GenBody Inc); Group C: Biocredit COVID-19 Ag (RapiGEN).

We assessed the LoD of five RDT by using a 10-fold serially diluted viral stock with known virus concentration. The PanbioTM COVID-19 Ag rapid test (Abbott) and the Standard Q COVID-19 Ag (SD Biosensor) RDT had the lowest LoD (1.74 × 10^5^ RNA copies/mL, assay group A). The RDT COVID-19 Ag Respi-Strip (Coris BioConcept) and GenBody COVID-19 Ag (GenBody Inc) had a LoD of 1.90 × 10^6^ RNA copies/mL (assay group B), and the Biocredit COVID-19 Ag (RapiGEN) RDT had a LoD of 2.82 × 10^7^ RNA copies/mL (assay group C).

We used the LoD of the RDT to estimate the proportion of infectious cases that would be detected if the different RDT were used as the first line of screening in the current test routine (Table). With the most sensitive RDT, assays in group A, 97.30% (range: 88.65–99.77) of infectious individuals would be detected. This decreased to 92.73% (range 60.30–99.77) and 75.53% (range: 17.55–99.77) for assays in group B and C, respectively. We repeated the analyses and stratified by days post onset with a subset of 1,450 specimens obtained from symptomatic individuals. Of samples obtained within 0–6 days post onset, 96.66% (range: 88.65–99.69) would be detected by assays in group A, 88.65% (range: 60.30–88.65) by assays in group B and 64.14% (range: 17.55–99.69) by the assay in group C (see Supplementary Table S1 for the complete results of this additional analysis). The time from sample collection to RT-PCR results was systematically recorded and those test results were available on the same day for 32.9% of specimens, on the next day for 55.3%, after 2 days for 11.2% and after 3 days for 0.6%.

## Discussion

The use of RDT for screening offers the potential for rapid identification of those individuals at greatest risk of spreading the infection [4]. We tested this line of reasoning based on real life data, as a basis for discussion on choices for assays and testing algorithms. The advantage of faster time to result and therefore initiation of contact tracing is a great added benefit of RDT. Our preliminary analysis suggests differences in the RDT regarding their suitability for tracking infectious cases. We showed that the most sensitive RDT included in this study (PanbioTM COVID-19 Ag rapid test (Abbott) and Standard Q COVID-19 Ag (SD Biosensor)) were capable of detecting 97.3% of infectious cases in the setting of drive-through testing. Assuming that the implementation of rapid tests will lead to reporting of the results on the same day, followed by contact by a public health official in all cases, the proportion of cases with optimal start of contact tracing (same day as testing) can increase from 32.9% to 75.5% when using the least sensitive assay, and to 97.3% for the most sensitive assays.

The shortening of testing delays is a critical determinant of success of a contact tracing strategy, and shortening from 3 to 1 days can push expanding outbreaks into suppression with a reproductive number below 1 [7,8]. Our analysis also shows, however, that antigen RDT differ substantially in their ability to detect infectious cases, therefore requiring careful validation before routine application. In our setting, people were tested relatively soon after onset of disease, when viral loads are at their peak, thus ensuring highest sensitivity of the RDT.

Another factor to be considered when choosing an RDT is the ability to collect leftover material for whole genome sequencing (WGS) for molecular characterisation and transmission chain analysis. We were able to successfully sequence leftover materials from the SD Biosensor assay using Nanopore sequencing (data not shown) [9]. The proportion of samples with successful WGS results and use of leftover specimens from other RDT remains to be determined. Alternatively, two swabs (one for the RDT and one for WGS) can be collected if leftover material from the RDT kit is not suitable for WGS.

Variants of concern (VOC) of SARS-CoV-2 have shown increased transmissibility, and by 3 February 2022, only 61% of the world population and 10% of the population in low-income countries have received at least one dose of COVID-19 vaccine [10-12]. Hence, the TTI strategy will stay an important public health measure to control the SARS-CoV-2 pandemic in countries with high levels of virus transmission.

A limitation of this study is our assumption that the ability to culture a specimen is a proxy for infectiousness of an individual. Culture methods are not standardised among laboratories and the sensitivity of the assay depends on the used protocol and freshness of specimens. We used fresh specimens to determine the relationship between viral load and culture success, but cannot exclude that other factors may have led to an underestimation of the number of infectious cases, and an overestimation of the ability of RDT to identify infectious cases in this study.

This study was performed in a period without known circulation of VOC. Although it has been shown that several RDT are able to detect the SARS-CoV-2 Alpha and Beta (Phylogenetic Assignment of Named Global Outbreak Lineages (Pangolin) designation B.1.1.7 and B.1.351) variants, monitoring the sensitivity of RDT for the detection of emerging VOC is needed to prevent false negative test results [13].

A more challenging application is the use of RDT to test vaccinated individuals or persons without symptoms, as a strategy to reopen society after lockdowns. Here, in the absence of knowledge of time since exposure, negative predictive values are difficult to assess and the risk of false negative results is higher than in symptomatic persons who may be using physical distancing.

## Conclusion

The most sensitive RDT detected infectious COVID-19 cases with high sensitivity and can substantially improve time to test result compared with RT-PCR. The RDT offer hope to improve epidemic containment by more rapid isolation and contact tracing of the most infectious individuals and are a promising alternative for RT-PCR in low- and high-income countries.

## Supplementary Data

178000 Supplement
