# Supplementary material for: From more testing to smart testing: data-guided SARS-CoV-2 testing choices, the Netherlands, May to September 2020
Source: Euro Surveill. 2022 Feb 24;27(8):2100702. doi: 10.2807/1560-7917.ES.2022.27.8.2100702 (PMC8874867; doi:10.2807/1560-7917.ES.2022.27.8.2100702)
Supplement: Supplement [file 21-00702_vanBEEK_Supplement.pdf]

This supplementary material is hosted by Eurosurveillance as supporting information alongside the article 'From more testing to smart testing: data-guided SARS-CoV-2 testing choices, the Netherlands, May to September 2020', on behalf of the authors, who remain responsible for the accuracy and appropriateness of the content. The same standards for ethics, copyright, attributions and permissions as for the article apply. Supplements are not edited by Eurosurveillance and the journal is not responsible for the maintenance of any links or email addresses provided therein.

Supplementary Figure S1

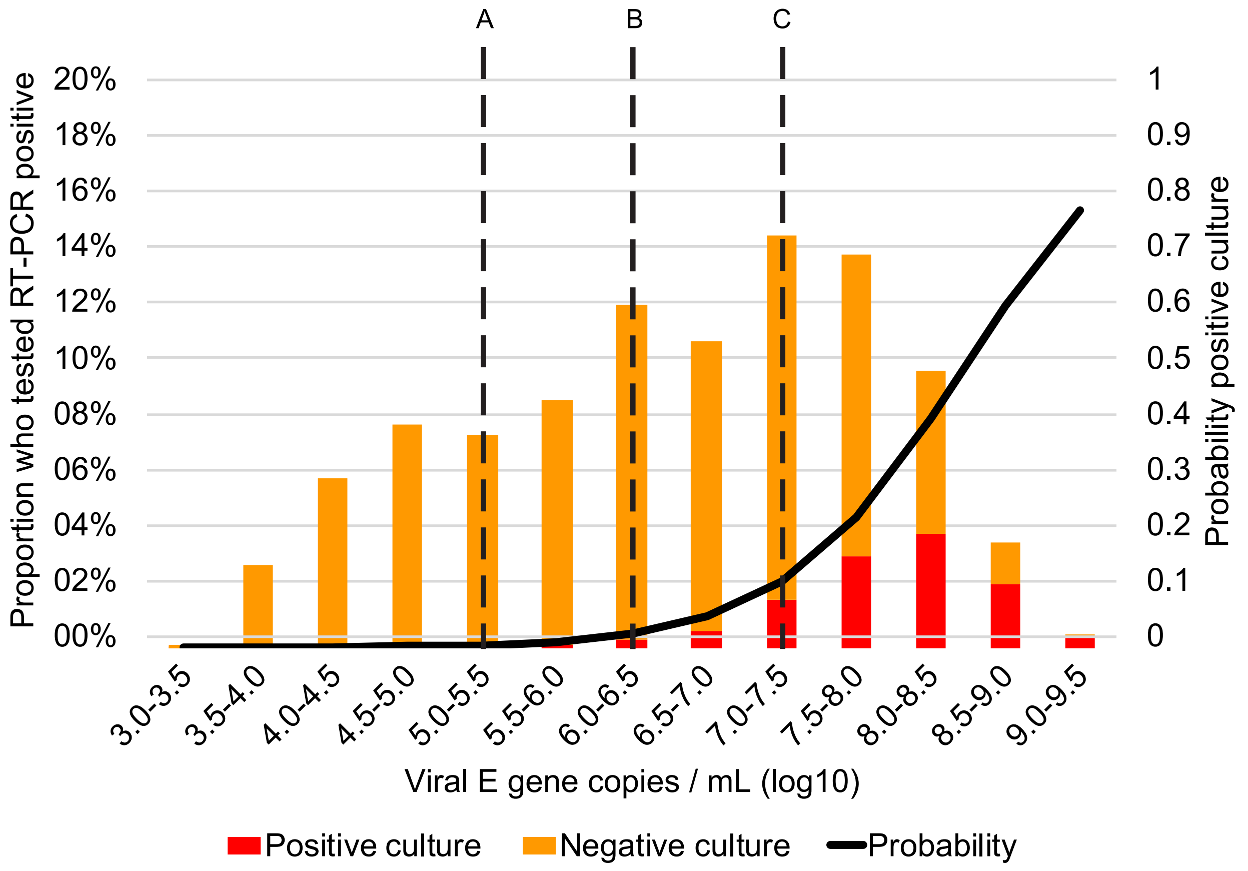

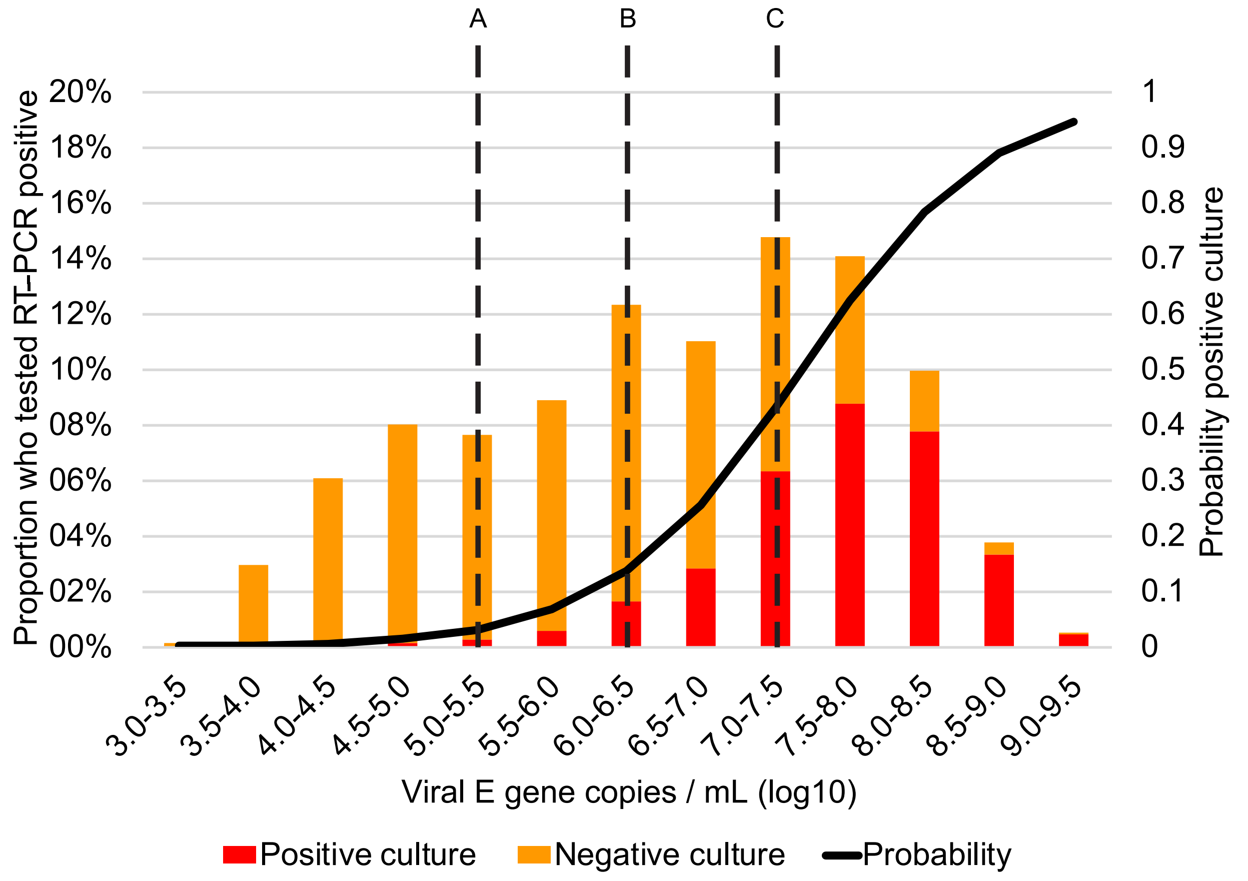

Supplemental Figure S1 Bars indicate distribution of viral RNA loads at time of diagnosis for 1,754 specimen of 1,739 community cases with RT-PCR confirmed SARS-CoV-2 infection presenting to a drive through test station. Black line denotes the projected probability of having infectious virus in the nose/throat swab as calculated using logistic regression based on **hospitalised severely ill patients** (Top) (van Kampen *et al.*, 2020) and based on **hospitalised patients with mild disease** (Bottom) (Wölfel *et al.*, 2020). Red bars indicate the predicted number of cases with shedding of infectious virus at time of diagnosis (positive culture), and orange bars indicate the number of predicted cases without shedding of infectious virus at time of diagnosis (negative culture). Dashed vertical lines indicate the limit of detection (LoD) of the different rapid detection tests (RDT's).
LoD A = PanbioTM COVID-19 Ag rapid test (Abbott), and Standard Q COVID-19 Ag (SD Biosensor);
LoD B = COVID-19 Ag Respi-Strip (Coris BioConcept), and GenBody COVID-19 Ag (GenBody Inc);
LoD C = Biocredit COVID-19 Ag (RapiGEN).

Supplementary table S1 *Estimated median, minimum and maximum proportion detected culture positive samples of 1,450 RT-PCR SARS-CoV-2 confirmed specimens with known days post onset (DPO) by rapid antigen tests with different detection limits.*

Results by DPO (<0 days, n = 31)

Rapid antigen assay group	Mild, outpatient median (min-max)	Hospitalised, mild median (min – max)	Hospitalised, severe median (min – max)
A	99.02% (94.07%-99.75%)	99.41% (97.54%-99.80%)	99.91% (99.61%-99.97%)
B	83.98% (61.59%-99.75%)	94.35% (86.90%-99.80%)	99.05% (97.56%-99.97%)
C	81.07% (37.52%-99.75%)	93.45% (75.52%-99.80%)	98.89% (94.73%-99.97%)

Results by DPO (0-6 days, n = 1096)

Rapid antigen assay group	Mild, outpatient median (min-max)	Hospitalised, mild median (min – max)	Hospitalised, severe median (min – max)
A	96.66% (88.65%-99.69%)	98.44% (95.79%-99.76%)	99.76% (99.32%-99.97%)
B	88.65% (60.30-88.65%)	95.79% (86.41%-99.76%)	99.32% (97.45%-99.97%)
C	64.14% (17.55%-99.69%)	87.84% (57.90%-99.76%)	97.76% (88.53%-99.97%)

Results by DPO (7 days or more, n = 323)

Rapid antigen assay group	Mild, outpatient median (min-max)	Hospitalised, mild median (min – max)	Hospitalised, severe median (min – max)
A	97.57% (88.65%-99.70%)	98.79% (95.79%-99.77%)	99.82% (99.32%-99.97%)
B	95.18% (60.30% - 99.70%)	97.91% (86.41%-99.77%)	99.68% (97.45%-99.97%)
C	91.12% (19.19%-99.70%)	96.57% (59.95%-99.77%)	99.45% (89.41%-99.97%)

Group A = Panbio™ COVID-19 Ag rapid test (Abbott)*, and Standard Q COVID-19 Ag (SD Biosensor);*
Group B = COVID-19 Ag Respi-Strip (Coris BioConcept), *and GenBody COVID-19 Ag (GenBody Inc);*
Group C = Biocredit COVID-19 Ag (RapiGEN).
